# Crowdsourcing Skin Demarcations of Chronic Graft-Versus-Host Disease in Patient Photographs: Training Versus Performance Study

**DOI:** 10.2196/48589

**Published:** 2023-12-26

**Authors:** Andrew J McNeil, Kelsey Parks, Xiaoqi Liu, Bohan Jiang, Joseph Coco, Kira McCool, Daniel Fabbri, Erik P Duhaime, Benoit M Dawant, Eric R Tkaczyk

**Affiliations:** 1 Dermatology Service and Research Service Department of Veterans Affairs Tennessee Valley Healthcare System Nashville, TN United States; 2 Department of Dermatology Vanderbilt University Medical Center Nashville, TN United States; 3 Department of Electrical and Computer Engineering Vanderbilt University Nashville, TN United States; 4 Department of Biomedical Informatics Vanderbilt University Medical Center Nasvhille, TN United States; 5 Centaur Labs Boston, MA United States

**Keywords:** graft-versus-host disease, cGVHD, crowdsourcing, dermatology, labeling, segmentation, skin, medical image, imaging, feasibility, artificial intelligence

## Abstract

**Background:**

Chronic graft-versus-host disease (cGVHD) is a significant cause of long-term morbidity and mortality in patients after allogeneic hematopoietic cell transplantation. Skin is the most commonly affected organ, and visual assessment of cGVHD can have low reliability. Crowdsourcing data from nonexpert participants has been used for numerous medical applications, including image labeling and segmentation tasks.

**Objective:**

This study aimed to assess the ability of crowds of nonexpert raters—individuals without any prior training for identifying or marking cGHVD—to demarcate photos of cGVHD-affected skin. We also studied the effect of training and feedback on crowd performance.

**Methods:**

Using a Canfield Vectra H1 3D camera, 360 photographs of the skin of 36 patients with cGVHD were taken. Ground truth demarcations were provided in 3D by a trained expert and reviewed by a board-certified dermatologist. In total, 3000 2D images (projections from various angles) were created for crowd demarcation through the DiagnosUs mobile app. Raters were split into high and low feedback groups. The performances of 4 different crowds of nonexperts were analyzed, including 17 raters per image for the low and high feedback groups, 32-35 raters per image for the low feedback group, and the top 5 performers for each image from the low feedback group.

**Results:**

Across 8 demarcation competitions, 130 raters were recruited to the high feedback group and 161 to the low feedback group. This resulted in a total of 54,887 individual demarcations from the high feedback group and 78,967 from the low feedback group. The nonexpert crowds achieved good overall performance for segmenting cGVHD-affected skin with minimal training, achieving a median surface area error of less than 12% of skin pixels for all crowds in both the high and low feedback groups. The low feedback crowds performed slightly poorer than the high feedback crowd, even when a larger crowd was used. Tracking the 5 most reliable raters from the low feedback group for each image recovered a performance similar to that of the high feedback crowd. Higher variability between raters for a given image was not found to correlate with lower performance of the crowd consensus demarcation and cannot therefore be used as a measure of reliability. No significant learning was observed during the task as more photos and feedback were seen.

**Conclusions:**

Crowds of nonexpert raters can demarcate cGVHD images with good overall performance. Tracking the top 5 most reliable raters provided optimal results, obtaining the best performance with the lowest number of expert demarcations required for adequate training. However, the agreement amongst individual nonexperts does not help predict whether the crowd has provided an accurate result. Future work should explore the performance of crowdsourcing in standard clinical photos and further methods to estimate the reliability of consensus demarcations.

## Introduction

Chronic graft-versus-host disease (cGVHD) is the leading cause of nonrelapse long-term morbidity and mortality in patients after allogeneic hematopoietic cell transplantation [1]. Skin is the earliest and most commonly affected organ [2]. Changes in cutaneous manifestations are used to evaluate treatment efficacy and disease progression, assessed by the affected surface area involvement. The National Institutes of Health (NIH) Skin Score is the primary outcome measure in major clinical trials. It is a composite score derived from assessment of the body surface area of erythema (categorical approximation), moveable sclerosis, and nonmoveable sclerosis, combined with a functional and symptomatic assessment [3,4]. The clinical trial for the first Food and Drug Administration–approved (2017) cGVHD treatment, ibrutinib, had a minimum of 25% body surface area erythema as an inclusion criterion [5]. In practice, surface area is estimated by visual assessment using either the Wallace rule of nines, Lund and Browder chart, or palmar units [6]. However, visual assessment of cGVHD suffers from low reliability. Mitchell et al [7] found that the threshold for defining change exceeding measurement error was 19%-22% of the entire body surface area for erythema. This poses a significant barrier to improving patient care through accurate tracking of disease severity and is compounded by the low availability of expert dermatologist evaluation [8].

A recent multicenter cohort study showed that for erythema-type cGVHD, percentage body surface area involvement was a better predictor of mortality than the categorical NIH cGVHD Skin Score [8]. Automated analysis of body surface area from photographs by artificial intelligence (AI) image analysis has shown promise, with a recent study finding that 77% of AI demarcations were scored as clinically acceptable by a board-certified dermatologist across more than 300 photos [9]. Further development of such engineering solutions is greatly hampered by the cost and difficulty of collecting expert demarcations for large numbers of photographs for training and validation.

Crowdsourcing data from a large number of nonexpert participants has been widely used for many medical applications [10,11], including bioinformatics [12], histology image labelling and cell segmentation [13-15], demarcating organs and regions of disease in both 2D and 3D radiology images [16,17], and combining crowd opinions with AI models for improving the severity scoring of diabetic retinopathy [18]. Recent work has also shown expert-level crowd performance for identifying some features of pigmented skin lesions in dermoscopic images, which comprise high magnification, narrow field of view cross-polarized photos of the skin surface [19].

Our study aimed to assess the ability of a crowd of nonexpert raters to demarcate photos of cGVHD-affected skin, which could provide a scalable solution for demarcating large numbers of patient photos for AI training. Cutaneous cGVHD often presents as complex areas of erythema and surface changes with ill-defined borders. This demarcation task typically requires significant training and is known to exhibit high variability even among experts [7]. To study the effect of training and feedback on crowd performance, we split raters into 2 groups (high and low feedback) that each received a different amount of ground truth feedback during data collection.

## Methods

### Materials

Patient characteristics are given in Table 1. The patient cohort had an age range of 21-72 (median 58, IQR 46-66) years and were photographed at 6 to 4520 days post–hematopoietic cell transplantation (median 1092, IQR 266-1724 days). Cutaneous cGVHD presented as erythema for 7 patients, sclerosis for 7 patients, and both erythema and sclerosis for 9 patients.

We took 360 3D photographs of the skin of 36 patients with cGVHD using a handheld commercial stereoscopic camera (Vectra H1, Canfield Scientific). This stereoscopic camera provides a cross-polarized flash and ranging lights to improve the consistency of photographic conditions between body sites and patients. It also enables accurate ground truth markings of affected skin areas directly on the 3D surface, which can generate multiple 2D views of the same skin from different angles to more closely emulate standard clinical photography. From each 3D photo, a set of 2D images were created from different viewing angles using Vectra Analysis Module software (Canfield) following an automated scripting protocol, as described in previous work [9,20]. Defining the original camera view as an angle of 0 degrees, we rotated the skin surface through combinations of 0 degrees, +15 degrees, and -15 degrees along the horizontal and vertical axes to create multiple views of the same skin area under identical photographic conditions. Table 2 shows how the photo set was split into photos for training and feedback (7 angles per photo) and photos for testing crowd performance (9 angles per photo). We refer to the training set as the “ground truth provided” set and the test set as the “ground truth withheld” set (Table 2). In total, the full set consisted of 3000 2D images for demarcation by the crowd, with the test set consisting of 711 ground truth withheld images with cGVHD-affected skin (per ground truth).

**Table 1:** Race, gender, ethnicity, and Fitzpatrick skin types of patients involved in the study.

Characteristic		Patients (n=36)
**Race, n (%)**		
	American Indian or Alaska Native	0 (0)
	Asian	0 (0)
	Black or African American	3 (8)
	Native Hawaiian or Other Pacific Islander	0 (0)
	White	33 (92)
**Gender, n (%)**		
	Man	26 (72)
	Woman	10 (28)
**Ethnicity, n (%)**		
	Hispanic or Latino	0 (0)
	Not Hispanic or Latino	36 (100)
**Fitzpatrick skin type, n (%)**		
	I	0 (0)
	II	8 (22)
	III	24 (67)
	IV	1 (3)
	V	1 (3)
	VI	2 (7)
Age (years), median (IQR)		58 (46-66)
Time since transplant (days), median (IQR)		1092 (266-1724)

**Table 2:** Distribution of photos and patients between the feedback (ground truth provided) and analysis (ground truth withheld) sets. For each stereoscopic photo, 9 images from different angles were produced.

Data category		Ground truth provided set		Ground truth withheld set
**cGVHD^a^-affected**				
	Photos (n=179), n (%)	100 (56)	79 (44)	
	Patients (n=25), n (%)	19 (76)	22 (88)	
**cGVHD-unaffected**				
	Photos (n=181), n (%)	20 (11)	161 (90)	
	Patients (n=23), n (%)	18 (78)	22 (96)	
**Total**				
	Photos (n=360), n (%)	120 (33)	240 (67)	
	Patients (n=36), n (%)	31 (86)	34 (94)	

^a^cGVHD: chronic graft-versus-host disease.

### Study Design

**Figure 1:**
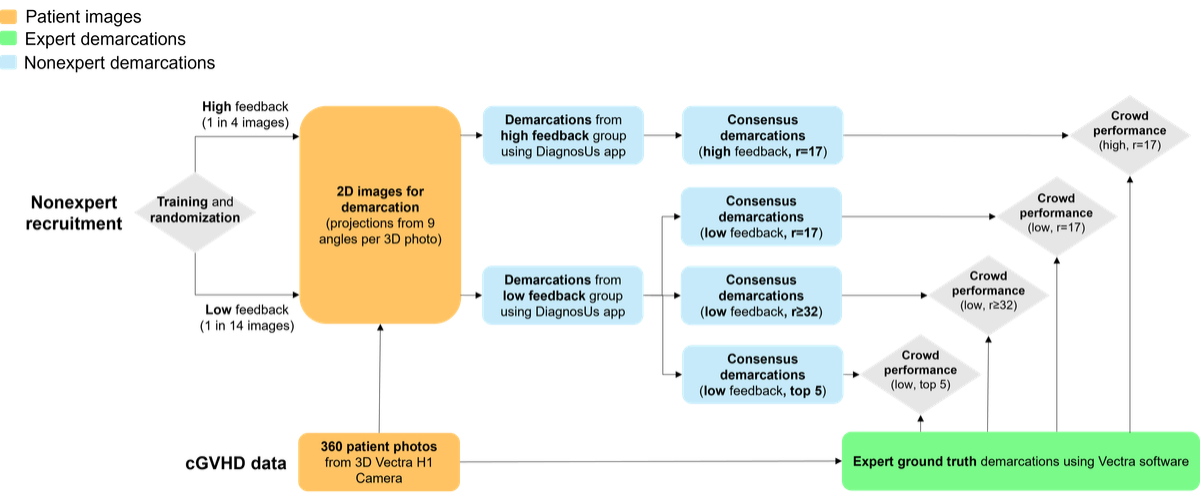
Flowchart of the study design. cGVHD: chronic graft-versus-host disease; r: rater.

A flowchart of the study design is shown in Figure 1. Patient photos were first demarcated by a trained expert to provide the ground truth. A crowd of nonexperts were then recruited through the Centaur Labs’ DiagnosUs app and randomized into low and high feedback groups after training. Crowd demarcations were gathered for each image, which were combined into separate consensus demarcations for each crowd. Performance was assessed by comparing the consensus demarcations to the expert ground truth.

### Ground Truth Demarcations

**Figure 2:**
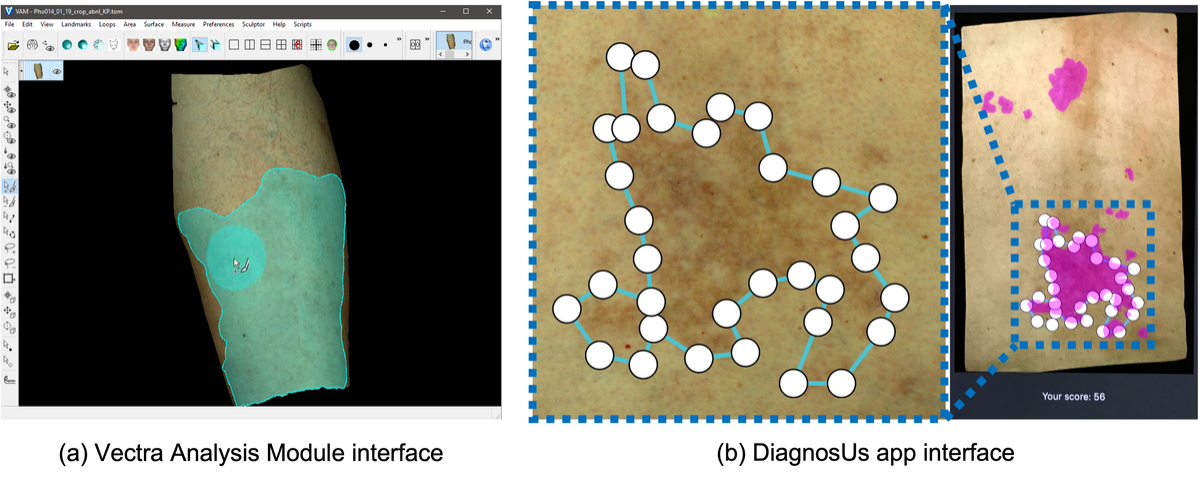
Annotation interfaces used for (A) ground truth demarcations using the Vectra Analysis Module and (B) crowd demarcations using the DiagnosUs app, including ground truth feedback during training.

A single expert (KP) provided ground truth demarcations of cGVHD-affected skin following an extensive training program [21], which were reviewed by a board-certified dermatologist (ERT) for accuracy. Affected skin areas were demarcated on the 3D skin surface using Vectra Analysis Module software (Figure 2a). The skin surface could be rotated and zoomed in 3D space, with affected skin areas demarcated using a paintbrush tool. This allowed us to create the most accurate demarcations of affected skin from the visual appearance alone. These ground truth areas were used for training the crowd through visual feedback and evaluating their performance.

### Crowd Training and Data Collection

The DiagnosUs app gamifies medical image demarcation tasks, creating time-limited competitions with leaderboards and prizes to incentivize engagement. Only 2D images are supported through the app and its mobile interface, necessitating the use of projected images of the skin surface. The interface uses the touch screen of a mobile device for demarcating areas using nodes, which outline the desired shape (Figure 2b). Multiple nontouching areas can be marked on a single image if needed, and node positions can be adjusted after placing before the final submission.

**Figure 3:**
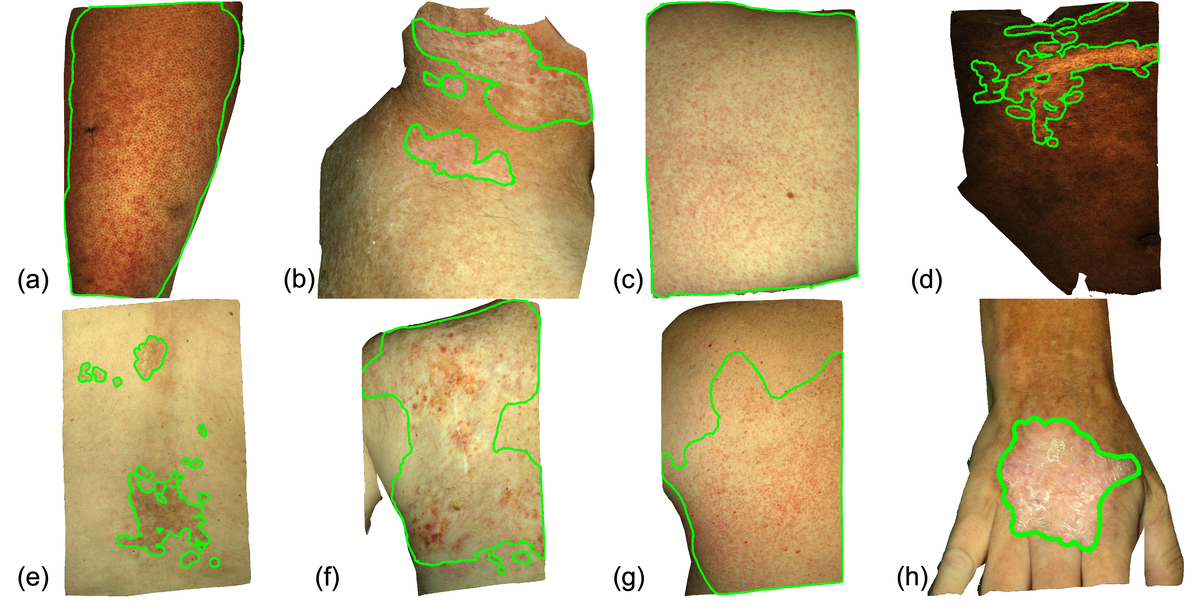
The 8 images used for training the crowd during study enrollment. Ground truth demarcations of cGVHD-affected skin are shown in green. The corresponding text descriptions of each disease presentation are given in Table 3. cGVHD: chronic graft-versus-host disease.

We used 8 images to train all raters before they began the demarcation task (Figure 3). For each training image, the rater was first shown the unmarked image with corresponding text by the expert dermatologist describing what features they should look for (Table 3). Upon submitting their demarcation, the rater was then shown the expert ground truth and their accuracy score. Training was completed once all 8 training images had been adequately marked. Each rater was randomly assigned to the high or low feedback group. No knowledge of the different groups or their assignments were communicated to the raters.

We held 8 24-hour competitions for data collection, with each user asked to demarcate 200 randomly selected images from the full set of 3000. Cash prizes were offered for each competition based on performance ranking, and all 200 images needed to be demarcated to be eligible for a given competition. Images were split into those for which the ground truth may be provided for feedback and quality assurance and those for which the correct answer was entirely withheld. Crowd performance was assessed on the 711 images of affected skin for which the ground truth was entirely withheld (never released to Centaur Labs). Raters in the high feedback group received feedback on 1 out of every 4 cases, while those in the low feedback group received feedback on 1 out of every 14 cases. For each image, we recorded the first 17 rater (r) opinions in both the high and low feedback groups, denoted as “r=17”. To test if a larger crowd could overcome the expected performance drop from less training feedback, data collection was extended for the low feedback group up to 32-35 demarcations per image, denoted as “r≥32”. Finally, the effect of tracking only the most reliable raters was examined. The performance of individuals was tracked on the images for which the ground truth was provided (ground truth provided set in Table 2), and the 5 best performers for each image in the ground truth withheld set were selected in the low feedback group, denoted as the “top 5” group.

**Table 3:** Text descriptions provided for each of the 8 training images shown in Figure 3.

Image^a^	Description
(a)	On the anterior left thigh, there are numerous diffuse erythematous macules and papules coalescing into patches and plaques. When large areas of the skin are affected by cGVHD^b^, visual comparison to healthy skin can be difficult.
(b)	On the right, posterior neck of a patient with sun damage, there is a large scaly, hypopigmented patch with erythematous borders.
(c)	On the back, there are diffuse, erythematous papules that coalesce into a plaque in the central, left portion of the image. This is an example of a morbilliform eruption, which can be caused by cGVHD. The entirety of the skin image may be affected by cGVHD, as in this example, so careful attention must be paid to all areas shown.
(d)	On the right upper quadrant of the abdomen, there is a well-defined hypopigmented patch. As skin tones vary between patients, visual comparisons should be made with unaffected regions on each image when possible.
(e)	On the back, there are several well-defined, hyperpigmented patches consistent with post-inflammatory changes that can occur in cutaneous cGVHD.
(f)	cGVHD may present as a large area of sclerosis with superimposed areas of color and texture changes.
(g)	On the back, there are numerous diffuse erythematous macules and papules. Areas of rash may be ill-defined, so care must be taken to examine the skin in detail. The annotation boundaries should be carefully placed to encompass all high certainty regions of affected skin.
(h)	On the dorsum of the right hand, there is a single well-defined, scaly erythematous plaque.

^a^Image letters correspond to the panels in Figure 3.

^b^cGVHD: chronic graft-versus-host disease.

### Crowd Consensus Demarcations

We constructed 4 different sets of consensus demarcations from the 4 constructed crowds (r=17 from the high feedback group and r=17, r≥32, and top 5 from the low feedback group). Each crowd’s consensus demarcation for an image was calculated by simple majority vote. For a given image, each pixel was labelled with the number of raters who marked it as affected. The consensus demarcation consisted of all pixels labelled by the plurality of raters (50% or more) in the crowd being analyzed, following the standard majority vote method [22]. The final consensus mask provides a binary label for every pixel in the image, being either cGVHD-affected skin or cGVHD-unaffected skin. The consensus demarcation for each crowd was considered their best estimate of cGVHD-affected skin for the given image.

### Agreement Measures

Crowd demarcations were analyzed only for photos for which the ground truth was never provided to any of the app users. We used 2 metrics: the Dice coefficient and the surface area error. To measure spatial overlap, we used the machine vision metric of the Dice coefficient [23], which ranges from 0 for no overlap to 1 for perfect agreement. For context, a recent study of 3 nonexperts who underwent an extensive 4-month training program for demarcating cGVHD led by a board-certified dermatologist were found to achieve a median Dice of 0.75 (IQR 0.68-0.84) when compared to the expert [21]. While commonly used for comparing demarcations in many medical imaging tasks, use of the Dice metric alone has been shown to be inadequate for capturing training effects for cGVHD skin demarcations [21]. Therefore, we also calculated the surface area error, which represents the absolute difference in the percentage of skin area marked by the crowd compared to the ground truth. For example, if the ground truth demarcation covered 10% of the skin area and the crowd marked 25%, then the surface area error is 15%. This performance measure of skin area estimation parallels the scoring accuracy measures often used for in-person clinical assessment [7].

### Learning Effects

The effect of feedback and experiential learning on the performance of the crowd was examined by tracking the performances of individual raters over the first 100 images with cGVHD-affected skin as the ground truth.

### Ethical Considerations

This study was performed using nonidentifiable photographs under Vanderbilt University institutional review board exemption 191042.

## Results

### Demarcating cGVHD-Affected Skin

Combined across all 8 competitions, a total of 291 raters were recruited, with 130 (45%) assigned to the high feedback group and 161 (55%) to the low feedback group, 111 (38%) of which contributed to the low r=17 crowd. This produced a total of 133,854 individual demarcations, including 54,887 (41%) from the high feedback group and 78,967 (59%) from the low feedback group.

**Figure 4:**
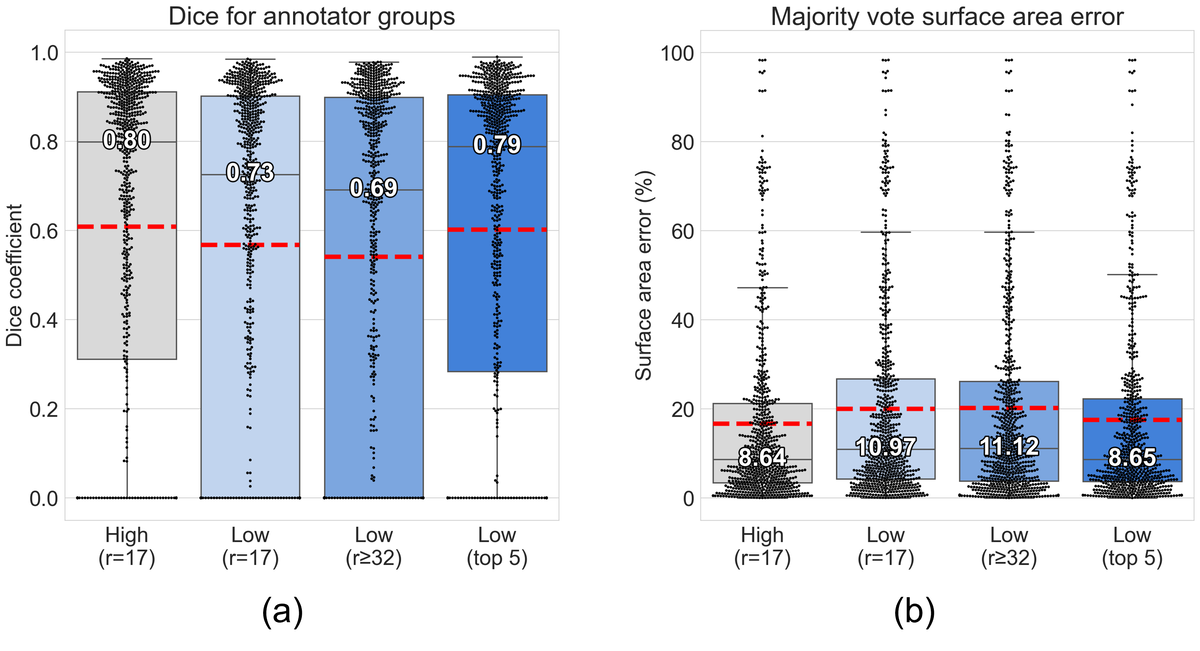
Performance of crowd groups for demarcating images with cGVHD-affected skin per ground truth using the (A) Dice coefficient and (B) surface area error. Each point represents the majority vote mask for a single image (711 images in total). Whiskers indicate 1.5 × IQR. Mean values are shown indicated by the dashed red line. cGVHD: chronic graft-versus-host disease; r: rater.

The subset of photos for which no ground truth was shown to any user included 79 photos from 22 patients with cGVHD-affected skin per the completely withheld ground truth (Table 2). To avoid the possibility that a user might have somehow seen the solutions to their delineation task for a particular patch of skin, we assessed the performance of the crowd on these 711 images (9 angles per photo) only. Figure 4a shows the crowd performance by Dice. The high feedback r=17 crowd and low feedback top 5 crowd were not significantly different according to a Mann-Whitney *U* test (*P*=.64), with a Dice coefficient of 0.8. Compared to the high feedback crowd, both the low r=17 and low r≥32 crowds were significantly different, with Dice coefficients of 0.7. Figure 4b shows the surface area error for each group. The high feedback and low feedback top 5 crowds had a surface area error of 9%, but the other 2 low feedback crowds were significantly different at 11%.

**Figure 5:**
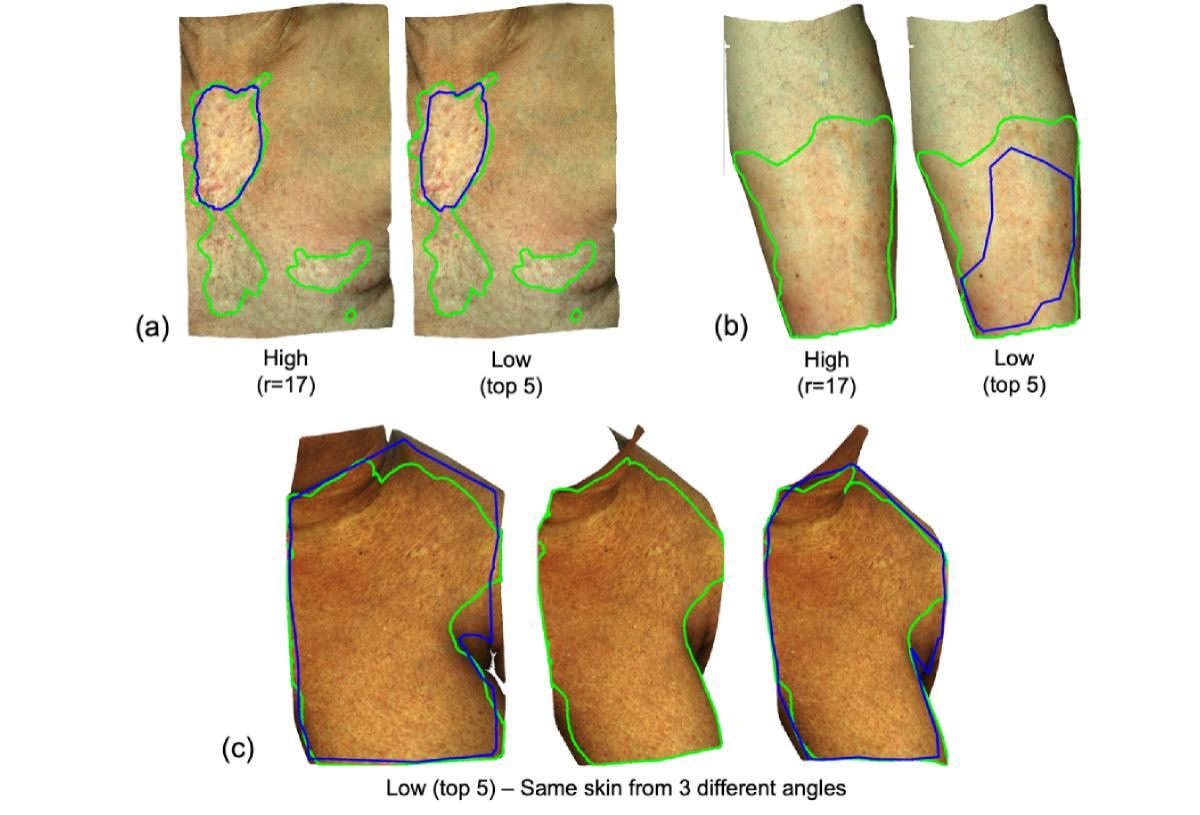
Example demarcations from the crowd (blue) versus ground truth (green). (A) Consistent demarcation of highly affected areas by crowds assembled from both the high and low feedback groups, but both missed areas of subtle surface changes. (B) The high feedback crowd failed to identify abnormal changes while low feedback top 5 crowd identified 75% of abnormal skin areas. (C) High variability between images of the same skin region viewed from different angles by the low feedback top 5 crowd. cGVHD: chronic graft-versus-host disease; r: rater.

Figure 5 shows examples of inconsistencies observed across the crowds. Figure 5a shows consistent demarcation of highly affected areas by both the high and low top 5 crowds; however, there was poor identification of subtle surface changes, which were also marked in the expert ground truth. Figure 5b demonstrates an instance where the high feedback crowd failed to identify abnormal changes while the low top 5 crowd identified 75% of the abnormal skin area. Figure 5c highlights an instance of high variability between images from different angles of same skin region in the low top 5 crowd, where 2 images show good agreement with the ground truth, but the third image consensus predicted no affected skin. In all cases, we also noted the sharp edge and lower specificity of areas marked by the crowd. This is likely due to the mobile interface providing lower fidelity for marking complex shapes as compared to the Vectra Analysis Module software used for marking the ground truth (Figure 2).

### Reliability of Demarcations

**Figure 6:**
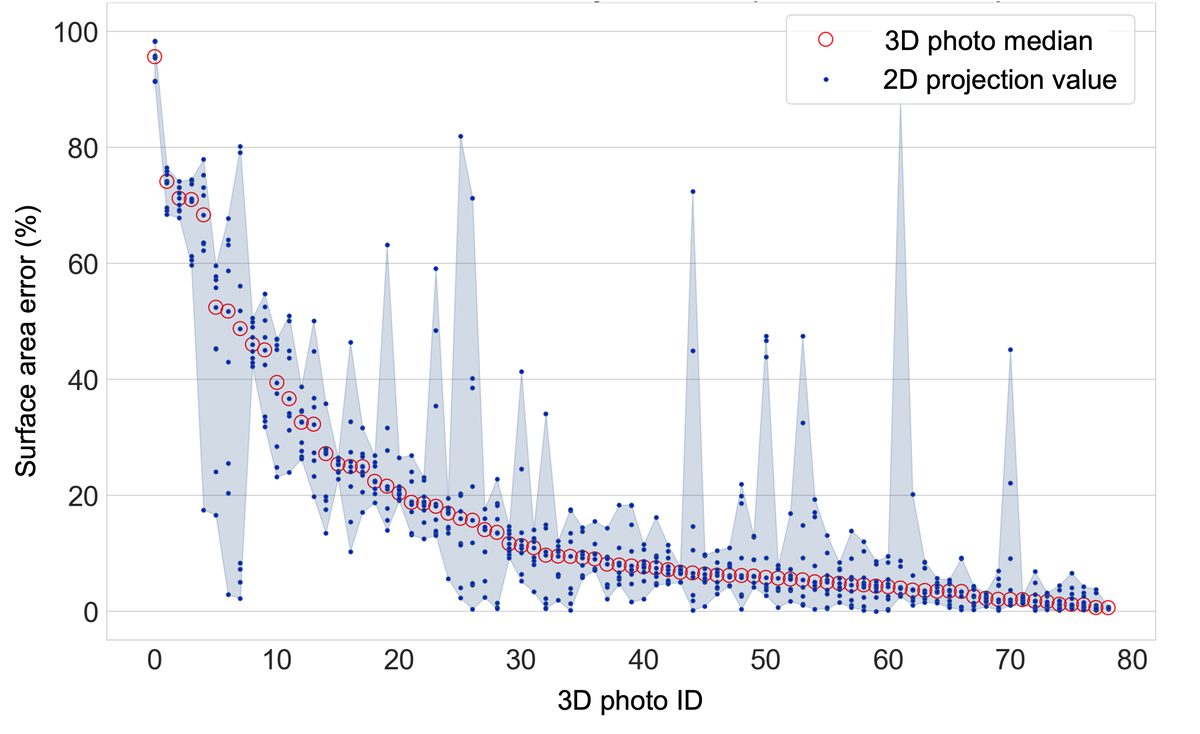
Per-photo surface area error for the low feedback group (top 5 raters). 3D photo IDs are ordered by decreasing median error. The shaded area shows the range of error between 2D projections for each 3D photo.

Despite good median performance of the different crowds across the full set, we observed a number of high error images in all groups (Figure 4b). Because the expert ground truth was available in 3D on our unique photo set but crowd annotation was done in 2D, we were able to test the performance of the crowd on the same area of skin under identical photographic conditions from different viewing angles. Figure 6 shows the surface area error for the low feedback top 5 crowd for each of the 9 projected images from each 3D photo, ordered by descending median error. We observe outlier images with significant errors in both high and low median error photos. The set of individual raters contributing to the consensus demarcation will vary between images, suggesting that interrater variability could contribute to the inconsistent reliability across images of the same skin.

**Figure 7:**
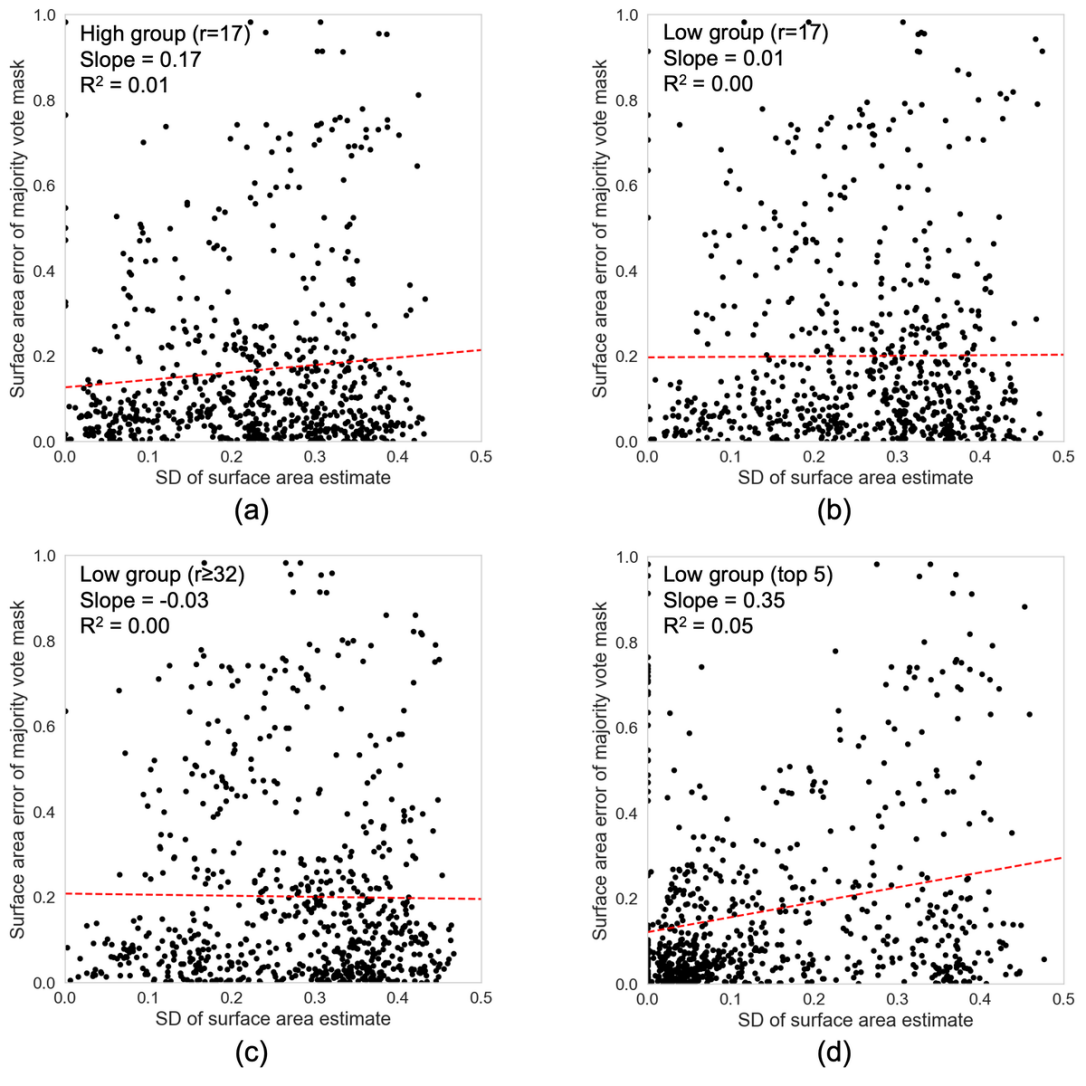
Surface area error of the majority vote mask versus the SD of surface area estimates for each photo. Slope and coefficient of determination (*R*_2_) for the linear regression fit (red dashed line) are also given.

To test if there was an observable association between the level of disagreement and the accuracy of the crowd demarcations, Figure 7 shows correlation plots of the surface area error of the consensus mask for a given photo against the SD of the crowd’s estimate of surface area for that photo. We found no significant correlation between the variability of individual raters and the accuracy of the consensus mask, with a near-zero coefficient of determination for simple linear regression in all groups. Therefore, the level of disagreement between raters cannot be used as a measure of reliability for this task.

### Performance Over Time

**Figure 8:**
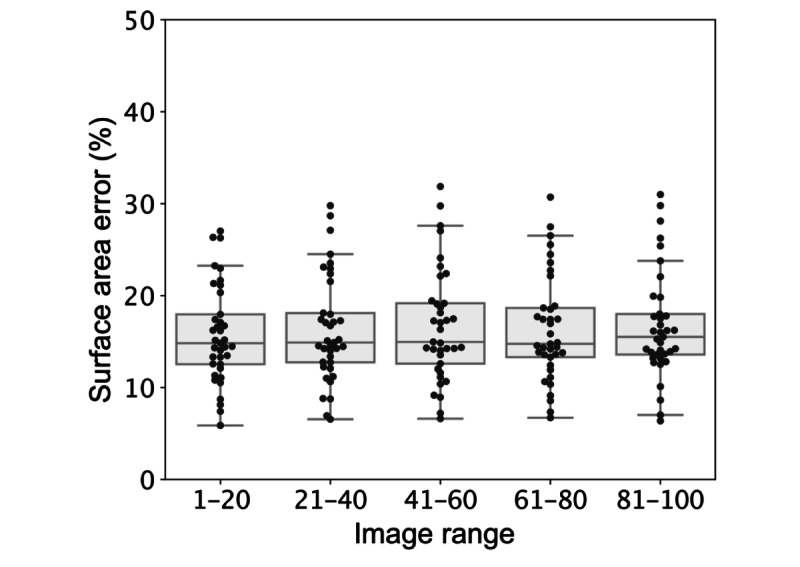
Surface area error of individual raters in successive groups of affected images. Error is displayed for all 37 raters who marked at least 100 affected images. Each point is the mean error for a given user for the group of 20 photos in the stated image range.

The effect of training on performance was examined by tracking the performance of all raters over the first 100 affected skin photos that they marked for which the ground truth was never shown. A total of 37 raters met this criterion. The mean performance over time for each eligible rater is shown in Figure 8, with no increase in performance seen over the first 100 images marked. Similar results on minimal training effects have been reported in other crowdsource studies [17].

## Discussion

### Principal Findings

A crowd of nonexpert raters was able to achieve good overall performance for segmenting cGVHD-affected skin with minimal training. The median surface area error was less than 12% for all crowds. The low feedback r=17 crowd performed slightly poorer than the high feedback crowd, with a 2% to 3% increase in surface area error. Recruiting more raters to the low feedback group for a larger crowd (r≥32) did not improve performance relative to the original low feedback crowd (r=17). However, tracking the top 5 most reliable raters from the low feedback group for each image was able to recover almost identical performance to the high feedback crowd. We believe this is due to individuals within the crowd likely having different skill levels for the assigned task, as has been noted in similar crowdsource studies [22]. We therefore recommend tracking rater performance to ensure the most reliable individuals contribute to the consensus demarcations. This optimal strategy will yield the best crowd performance while lowering the required number of expert demarcations for training.

High variability between different angles of the same skin area were observed, raising concerns for the reliability of the consensus demarcation for any given image with an unknown ground truth. Higher variability between raters for a given image was not found to correlate with lower performance of the crowd consensus demarcation and cannot therefore be used as a measure of reliability. Finally, no significant learning was observed during the task as more photos and feedback were seen.

### Limitations

A limitation of our study was the lack of diversity in skin types. Our cohort was dominated by lighter skin tones; 32 patients had Fitzpatrick skin type III or lower, while only 4 patients had skin types IV and higher. Despite the good overall performance of the crowd, further study is needed to disentangle the possible sources of disagreement that were observed and develop methods to mitigate these effects.

### Future Work

We have demonstrated the potential utility of employing a crowd of nonexpert raters for demarcating cGVHD-affected skin in patient photos. Next steps should explore if this performance is maintained when applied to standard clinical photos, where lighting conditions, imaging distances, and photo quality may be more variable than the more standardized set used here. In addition, further methods of estimating the reliability of the consensus demarcations should be explored to provide more robust quality assurance and filter out high error outliers. Future studies using more extensive training techniques and a unified interface for crowd and expert demarcations will also be important for establishing nonexperts’ understanding of the complex task and methods to minimize potential sources of variability. Accurately recognizing active disease is a major clinical concern, with even expert dermatologists commonly disagreeing on whether a skin area is erythematous or hyperpigmented [24]. New technologies, such as hyperspectral imaging, have shown promise for addressing this limitation in clinical practice [25]. The ability of the crowd to differentiate between disease types should also be explored in future studies given the good overall performance for recognizing cGVHD-affected skin.

Ultimately, the application of crowdsourcing could offer a scalable solution for labelling large sets of images for the training of automated AI algorithms. The effect of training with a larger volume of lower quality demarcations versus a smaller number of expert demarcations, as reported previously [9], also warrants future investigation.

### Conclusion

We have shown that a crowd of nonexpert raters is capable of delineating surface areas of cGVHD-affected skin (9%-11% surface area error) better than the current clinical standard (19-22% [7]). Crowd demarcation therefore offers a practical solution for accurately demarcating large databases of patient photos, which is a crucial unmet need for training reliable AI image analysis methods. Such methods could provide a clinically meaningful improvement to the current standard given that body surface area has been shown to be a better predictor of mortality than the NIH cGVHD Skin Score [8].

## Abbreviations

AI: artificial intelligence
cGVHD: chronic graft-versus-host disease
NIH: National Institutes of Health
r: rater
